# Artificial Intelligence-Augmented Advancements in the Diagnostic Challenges Within Renal Cell Carcinoma

**DOI:** 10.3390/jcm14072272

**Published:** 2025-03-26

**Authors:** Mladen Doykov, Stanislav Valkanov, Usman Khalid, Jasmin Gurung, Gancho Kostov, Bozhidar Hristov, Petar Uchikov, Maria Kraeva, Krasimir Kraev, Daniel Doykov, Katya Doykova, Siyana Valova, Lyubomir Chervenkov, Stefan Konsulov

**Affiliations:** 1 Department of Urology and General Medicine, Medical Faculty, Medical University of Plovdiv, 4002 Plovdiv, Bulgaria; stanislav.valkanov@mu-plovdiv.bg; 2 Medical Faculty, Medical University of Plovdiv, 4002 Plovdiv, Bulgaria; usmankhalid957@gmail.com (U.K.); jasmingurung12@gmail.com (J.G.); 3 Department of Special Surgery, Faculty of Medicine, Medical University of Plovdiv, 4002 Plovdiv, Bulgaria; gancho.kostov@mu-plovdiv.bg (G.K.); petar.uchikov@mu-plovdiv.bg (P.U.); 4 Second Department of Internal Diseases, Section “Gastroenterology”, Medical Faculty, Medical University of Plovdiv, 4002 Plovdiv, Bulgaria; bozhidar.hristov@mu-plovdiv.bg (B.H.); daniel.doykov@mu-plovdiv.bg (D.D.); 5 Department of Otorhinolaryngology, Medical Faculty, Medical University of Plovdiv, 4002 Plovdiv, Bulgaria; maria.kraeva@mu-plovdiv.bg (M.K.); stafan.konsulov@mu-plovdiv.bg (S.K.); 6 Department of Propedeutics of Internal Diseases, Medical Faculty, Medical University of Plovdiv, 4002 Plovdiv, Bulgaria; krasimir.kraev@mu-plovdiv.bg; 7 Department of Diagnostic Imaging, Medical Faculty, Medical University of Plovdiv, 4002 Plovdiv, Bulgaria; katya.doykova@mu-plovdiv.bg (K.D.); lyubomir.chervenkov@mu-plovdiv.bg (L.C.); 8 Second Department of Internal Diseases, Section “Nephrology”, Medical Faculty, Medical University of Plovdiv, 4002 Plovdiv, Bulgaria; siyana.valova@mu-plovdiv.bg

**Keywords:** artificial intelligence, diagnostics, renal cell carcinoma, histology, multi-omics, imaging, perioperative diagnostics

## Abstract

**Background:** Advancements in artificial intelligence (AI) diagnostics for renal cell carcinoma (RCC) provide valuable information for classification and subtyping, which improve treatment options and patient care. RCC diagnoses are most commonly incidental due to a lack of specific characterizations of subtypes, often leading to overtreatment. Accurate diagnosis also allows for personalized patient management. Different diagnostic methods, such as histopathology, multi-omics, imaging, and perioperative diagnostics, show a lot of promise for AI. **Objective:** This literature review focuses on developments in RCC diagnostics and their outcomes, efficacy, and accuracy in classification. **Method:** We conducted a non-systematic review of the published literature to explore advancements in the diagnostics of RCC. The PubMed and Google Scholar databases were reviewed to extract relevant information. The literature shows that AI can help distinguish RCC from other kidney lesions and track tumor growth. The integration of radiomic features with clinical metadata further enhances the results. This enables clinicians to implement personalized treatment plans. The application of artificial intelligence in perioperative diagnostics enhances decision-making, improves patient safety, mitigates intraoperative complications, and accelerates recovery. Alongside the advancements in AI-assisted diagnostics, there are problems that need to be addressed, including selection bias, demand for larger and diverse datasets, and reliable validation. **Conclusions:** Despite the challenges, using AI to help with RCC diagnosis could lead to better patient outcomes, a new standard of care for RCC patients, and more personalized cancer management for each patient.

## 1. Introduction

Renal cell carcinoma (RCC) represents more than 90% of kidney cancers and is considered the most fatal urogenital cancer, with a mortality rate of 30–40%, as opposed to bladder and prostate cancer, which only account for a 20% mortality rate. The histopathological spectrum of RCC is based on molecular genetics and morphologic features. Clear-cell RCC, papillary RCC (types I and II), and chromophobe are the most common solid RCC types established on histological features, accounting for 70–90% of cases. RCC is a complicated and varied illness that can present in many different ways, from pain, blood in the urine, and lumps that can be felt to side effects like polycythemia, cachexia, weight loss, and fever. Some cases involving small masses may remain asymptomatic. Therefore, only 30% of patients with RCC receive a diagnosis based on their symptoms.

A considerable proportion of renal cell carcinoma (RCC) diagnoses are incidental, and the recent increase in RCC incidence is due to routine imaging performed for other medical conditions [1,2]. The majority of identified renal lesions are small and benign, primarily consisting of renal cysts, whereas angiomyolipomas and oncocytomas comprise a small fraction [3]. Pre-therapeutic classification is essential for the development of emergent and innovative therapeutics. Surgical resection is the standard therapeutic approach for localized renal cell carcinoma (RCC), with nephron-sparing surgery typically employed to preserve renal function. Notwithstanding surgical intervention, 10% to 17% of kidney tumors are subsequently determined to be benign following comprehensive histopathological assessment. Consequently, accurate diagnostics are necessary for appropriate treatment. Active surveillance is increasingly being recognized as a viable approach for managing small renal masses, particularly among elderly patients and those with comorbidities who face heightened risks associated with surgical interventions. Prior to active surveillance, a renal mass biopsy (RMB) is conducted to analyze the histopathological subtype. Staging provides further confirmation before therapeutic interventions [2].

However, the use of RMB is controversial because it poses different risks, such as bleeding, tumor seeding, and the development of perinephric hematomas [4]. In a systematic review and meta-analysis by Marconi et al., data from 57 studies showed low-rate hematomas (4.3%) and a rare incidence of severe bleeding (0–1.4%) [5]. RMB achieved a sensitivity and specificity of 99.1% and 99.7%, respectively, in the diagnostic accuracy of detecting malignancy. However, only 64.6% of oncocytomas diagnosed by RMB were confirmed benign after histopathological evaluation following surgical resection in a study by Moch et al. [6]. This shows the need for improvements in diagnostic methods to increase accuracy for adequate disease management while reducing invasiveness [2]. Artificial intelligence (AI) is the capability of machines to perform tasks autonomously and find solutions without specific programming for each action. Currently, the medical system has experienced a surge in AI adoption due to its accessibility, even among individuals without a strong background in mathematics or statistics. However, the algorithm it primarily operates on, such as deep learning (DL), is difficult to understand, posing a serious challenge for clinical use.

The foundational layer of the algorithm requires data. The utilization of paper charts, analog radiographs, and light microscopes to aid patient treatment has been replaced by superior modern technologies such as digitized technology, electronic health records, and virtual pathology. This transition to electronic records has produced enormous amounts of data that can be harnessed in AI and data-characterization algorithms [7]. AI has the potential to facilitate data analysis and reveal patterns that may not be perceptible to the human eye.

Machine learning (ML) is a subset of AI that aids in creating foundational elements by learning from digital images of tissue samples. ML plays a vital role in histopathology, enabling the analysis of tissue samples with the ability to accurately identify and classify various sections within a tissue sample. The capabilities of ML have been significantly improved with the advancement of deep learning (DL), which utilizes artificial neural networks composed of multiple layers inspired by the communication patterns of biological neurons. DL models have the capacity to analyze extensive and varied data and extract valuable insights, such as in cancer diagnostics.

The purpose of this non-systematic review was to examine recent advancements in artificial intelligence-enhanced diagnostic methodologies for renal cell carcinoma (RCC).

## 2. Materials and Methods

**Figure 1:**
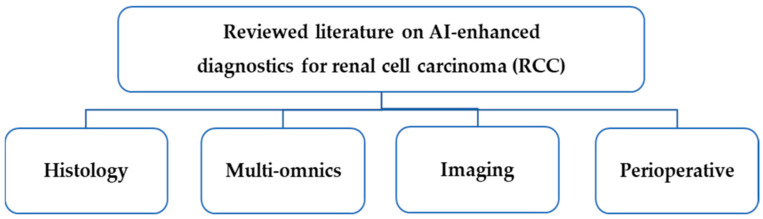
Areas of AI-enhanced diagnostics for RCC identified in the reviewed literature.

In this article, we present the results of a literature review on emerging AI-based models for RCC diagnostics in four areas of use: histological analysis, multi-omics, imaging, and perioperative planning. Figure 1 summarizes four areas of AI-enhanced diagnostics for RCC that were identified in the reviewed literature.

To identify relevant sources on advancements in AI-augmented diagnostic approaches to renal cell carcinoma, we searched the PubMed and Google Scholar databases. Search keywords were used to find relevant studies published up to 15 January 2025. The search terms included artificial intelligence, diagnostics, renal cell carcinoma, histology, multi-omics, imaging, and perioperative diagnostics. Only articles matching our search terms were considered appropriate. Articles published in languages other than English were not selected.

The sources were analyzed based on the following inclusion criteria: (1) publications had to discuss artificial intelligence in relation to renal cell carcinoma; and (2) publications had to be full-text original articles and/or review articles that included all necessary subsections, such as introduction, materials and methods, results, and discussion with conclusions.

Preliminary screening was conducted by examining the titles and abstracts, subsequently followed by a comprehensive review of the selected full-text articles. We augmented our dataset by identifying pertinent articles from the reference listings of those already incorporated into our collection. A deliberation among the authors facilitated the refinement of the selected articles to identify those of the utmost relevance.

## 3. Areas of AI-Enhanced Diagnostics for Renal Cell Carcinoma

### 3.1. Histology

Histopathology is crucial in renal cancer diagnostics, providing a reliable and comprehensive system for identifying and classifying cancer cells into their appropriate stages. The use of AI to record, analyze, and classify cellular patterns could completely change the field of histopathology, making diagnosis more accurate and faster. One major issue with manual pathology as a diagnostic tool is that it does not evaluate nuclear grading and spatial structures in enough detail to help us understand the different tissue structures that determine the biological and clinical states of renal cancer.

In response to this issue, Nyman et al. devised a deep learning framework that employs ResNet50, a convolutional neural network (CNN). This framework generates quantitative high-resolution whole slide images (WSIs) of clear cell renal cell carcinoma (ccRCC), thereby facilitating the systematic identification of established pathological features, including tumor tissue and nuclear grade. By generating spatial maps and regional adjacency graphs from a single whole slide image, the CNN identifies intratumor heterogeneity features beyond manual review. Graph-based microheterogeneity improves prognostic information, going beyond standard pathology scores and predicting how immune checkpoint inhibitors will work in clinical settings [8].

Holdbrook et al. created a pipeline that objectively and consistently measures nuclear pleomorphic patterns and helps pathologists make accurate decisions about the histopathology of ccRCC. When applied to a cohort of 59 patients, the automated learning program identified and analyzed prominent nucleoli, classifying the histopathologic images as low or high grade. A five-fold cross-validation of the single-patch classifier showed a strong correlation (r = 0.59) between predicted feature values and regional multigene scores [9].

In a related study, Abu Haeyeh et al. sought to differentiate between malignant renal cell carcinoma and benign tumors while simultaneously distinguishing tumor subtypes. This was accomplished through the integration of decision-fusion methodologies to ensure precise data classification. The dataset comprised four categories of renal tissues: non-renal cell carcinoma (non-RCC) renal parenchyma, non-RCC adipose tissues, clear cell renal cell carcinoma (ccRCC), and clear cell papillary renal cell carcinoma (ccpRCC). The deep learning system produced promising outcomes, attaining an accuracy of 93.0%, a sensitivity of 91.3%, and a specificity of 95.6% in distinguishing clear cell renal cell carcinoma (ccRCC) from chromophobe renal cell carcinoma (chRCC) or non-renal cell carcinoma (non-RCC) tissues. Furthermore, the methodology demonstrated superior performance in the classification of renal cell carcinoma (RCC) subtypes compared to the ResNet-50 model [10].

To classify kidney WSIs, Abdeltawab et al. created a pyramidal deep learning pipeline incorporating three CNNs to curate pixel-wise and patch-wise classifications. In addition to surpassing the performance of other neural networks, including ResNet, the method complements the pathologist’s expertise in automated histopathological diagnosis [11].

An independent study assessed the efficacy of three models in forecasting tumor mutation burden and the status of VHL mutations. A self-supervised attention-based multiple instance learning (SSL-ABMIL) model was developed utilizing hematoxylin and eosin-stained histopathological images. Two distinct population groups were identified based on high and low tumor mutational burden (TMB), with a cut-off value of 0.9. The Wang-ABMIL model generated AUROC scores of 0.83 for TMB prediction and 0.8 for VHL mutation prediction. By leveraging the attention weights and prediction scores generated by the ABMIL model, attention heat maps demonstrated the model’s focus on tumor regions for high-TMB patients, reflecting a marked stromal lymphocytic infiltration for VHL mutation prediction. The results substantiate the efficacy of SSL-ABMIL models in forecasting VHL and TMB mutations predicated on histological characteristics. They assert their capability to integrate molecular biology with tumor morphology [12].

A study by Cai et al. addressed the scarcity of medical data banks and the benefits of texture representation by integrating texture feature descriptors with deep learning platforms. The proposed model achieved its best performance with an SVM classifier, reaching an accuracy of 98.54% when combining Alex-Net with Gabor filter features. Other feature combinations also delivered high accuracy results: 93.76% for Alex-Net with HOG, 94.52% with GLCM, 93.45% with LBP, and 97.39% with MRF. Integrating AlexNet with five texture descriptors enhanced classification accuracy. The system demonstrated superior performance compared to other models in mitigating deficiencies associated with cancer detection and the scarcity of medical data. This presents a promising solution for the precise and automated diagnosis of renal malignancy [13].

In contrast to the standardized application of neural networks for the detection of morphological patterns, He et al. explored the potential of integrating numerical data of specific marker proteins, including essential autophagy proteins (ATGs), obtained from immunohistochemical (IHC) images of RCC. Using IHC staining and automated quantification with RCC tissue microarrays, significant reductions in ATG1, ATG5, and LC3B levels were observed, indicating decreased basal autophagy in RCC. The application of K-Nearest Neighbor (KNN) machine learning identified LC3B as a strong indicator for ccRCC. High ROC curve values demonstrated the potential of p62 to serve as a robust marker for distinguishing chromophobe RCC subtypes. Combinations of autophagy-related proteins further differentiated RCC subtypes from normal tissue, thereby enhancing their potential as an adjunctive tool for the classification of RCC and for broader applications in precision oncology [14].

Table 1 summarizes the reviewed literature on AI applications in histology for RCC diagnostics.

### 3.2. Multi-Omics

Current practice provides a limited ground for the development and expansion of multi-omics and its utility in renal cancer diagnosis. This restricts its real-world application due to its minuscule involvement in clinical diagnostics. Despite this, the advent of multi-omics technologies built upon high-throughput sequencing, such as epigenome profiling, whole-genome DNA sequencing, and whole-transcriptome profiling, has the potential to shift dynamic practices shortly [15]. The heterogeneous molecular profiles and asymptomatic early stages of ccRCC amplify the need for novel diagnostic biomarkers. Jagga et al. built on this idea by using four supervised learning algorithms—J48, RF, SMO, and Naive Bayes—to differentiate between early-stage and late-stage ccRCC based on transcriptomic signatures using fast correlation-based feature selection. They trained their classification models on sequencing-based gene expression data from RNAseq experiments that they got from The Cancer Genome Atlas. The random forest prediction model outperformed the others in the study, with 89% sensitivity, 77% accuracy, and an AUC of 0.8. It predicted 62 genes that were different between the early-stage and late-stage groups, reinforcing the concept of molecular mechanisms with regard to disease progression and diagnostics [16].

Liu et al. explored the application of bioinformatics and neural networks for biomarker identification in ccRCC. Using information from the gene expression omnibus database, they initially identified differentially expressed genes (DEGs) that set ccRCC apart from healthy renal tissue. Ten hub genes were identified, including TPX2, AURKB, NCAPG, and CCNA2, from a network of protein interactions from the DEGs using cytoHubba. These genes demonstrated a high degree of expression, coinciding with poor overall survival rates. RT-qPCR validation confirmed that the expression levels of these hub genes aligned with public data in the neural network model. The strong correlations between CCNA2, AURKB, NCAPG, and TPX2 substantiated their roles as significant diagnostic biomarkers [17].

RCCs display altered cellular metabolism, which, in combination with their proximity to the urine, lays the groundwork for urine metabolomic profiling for assay development. Using the data acquired from liquid chromatography–mass spectrometry and nuclear magnetic resonance, it was fed into the optimally tuned machine learning platform. The potential utility of urine assay for RCC detection in clinical settings was supported by an AUC of 0.98, an accuracy of 88%, a sensitivity of 94%, and a specificity of 85% [18].

The popularity of liquid biopsy as a non-invasive diagnostic tool is making it a suitable candidate for the diagnosis of RCC. It is possible to get a real-time picture of the tumor’s genetic landscape by looking at tumor DNA, circulating tumor cells, and other biomarkers found in body fluids like blood. This allows not only for early detection but also for tracking disease progression and treatment response. AI optimization holds a lot of promise for personalized medicine in RCC because it enables clinicians to plan treatments that are specific to each patient’s tumor based on its molecular profile [19].

Through a fusion of artificial intelligence and lipidomics, multivariate models based on support vector machines yielded two individual lipid panels for ccRCC diagnosis and detection. With a 16-lipid panel discriminating ccRCC from controls with 95.7% accuracy in training and 77.1% in testing, a second model distinguished early- from late-stage ccRCC by achieving an accuracy of 82.1%. The lipids 3α-hydroxy-5α-androstan-17-one 3-sulfate, cis-5-dodecenoic acid, arachidonic acid, cis-13-docosenoic acid, PI(16:0/18:1), PC(16:0/18:2), and PC(O-16:0/20:4) demonstrated a potential to differentiate between early- and late-stage ccRCC [20].

Iwamura et al. further developed the integration of AI-optimized liquid biopsies when analyzing N-glycan signatures from one hundred serum subjects with RCC. Through a supervised machine learning model, the data fed to the program established a scoring system, assessing the probability of RCC. With an AUC of 0.99, a sensitivity of 90%, and a specificity of 99%, the RCC score maintained outstanding diagnostic performances. The detection of RCC across all pathological stages further underscored its reliability as a biomarker for early differentiation and diagnosis [21].

A challenge also lies in the differentiation of renal cell carcinoma from renal oncocytoma, mainly due to their morphological and histological similarities. Radiomic biopsies (RBs) provide an alternative outlook to a potentially more refined and definitive diagnosis. A comparison of random forest and AdaBoost binary classifiers on radiomic features extracted from two rounds of RB assessment was conducted. It was found that the best classifiers had an average AUC of 0.71 ± 0.024, and the RB round did not have a significant effect (*p* = 1.00). Moreover, there was no indication of performance degradation when testing on data from the alternate RB round (*p* = 0.85). Feature clustering revealed seven distinct clusters in each RB round with high consistency (Rand index = 0.981, *p* < 0.001). The results show that a stable radiomic signature from RB rounds can be derived to help differentiate between oncocytomas and chromophobe renal cell carcinoma [22].

Brennan et al. incorporated methylomics through profiling DNA methylation in fresh-frozen oncocytoma and chRCC tumors, as well as in the surrounding healthy tissue. Subsequently, using machine learning, it could identify signatures of differentially methylated cytosine–phosphate–guanine sites (CpGs) that robustly distinguish oncocytoma from chRCC. The model based on 30 CpGs successfully differentiated oncocytoma from chRCC in 10-fold cross-validation with an AUC of 0.96. It also distinguished chRCCs derived from the Cancer Genome Atlas from a separate set of oncocytomas (AUC = 0.87) and separated oncocytomas from other RCC subtypes and normal tissue, thereby establishing it as a reliable standalone biomarker for oncocytoma diagnosis [23]. A summary of the reviewed research on AI applications in multi-omics technology for RCC diagnostics is provided in Table 2.

### 3.3. Imaging

AI methods can be employed to identify patterns and biomarkers in multimodality imaging, including computed tomography (CT), magnetic resonance imaging (MRI), and positron emission tomography/computed tomography (PET/CT). In clinical practice, CT scans remain the basis for accurate diagnosis. At the same time, the application of AI expands the opportunities for further collaboration and refinement. Xu et al. used the stochastic gradient descent (SGD) algorithm with cross-entropy loss to pre-train deep learning models for 60 epochs. To further improve predictive accuracy, four different deep learning (DL) networks with individual weights were separately developed and tasked with 9978 images in the development cohort. The single deep learning model achieved an AUC of 0.864 in the validation cohort, while the ensembled model demonstrated superior performance with an AUC of 0.882. The results showed that artificial intelligence can offer accuracy similar to traditional biopsy methods while also being non-invasive and quick [24].

Research about the integration of AI with clinical metadata and CT scans has shown their joint capacity to reduce surgical ambiguity by providing a comprehensive grading of cancer patients. The results demonstrated an accuracy of 85.66%, precision of 84.18%, and an F1-score of 84.92%. Moreover, a higher level of accuracy was achieved in selecting surgical procedures for malignant RCC tumors, with an accuracy of 90.63%, precision of 90.83%, and an F1-score of 90.50%. Feature ranking identified tumor volume and cancer stage as key factors in determining patient diagnosis and treatment, allowing for more personalized patient management plans [25].

Certain variants of angiomyolipoma (AML) that lack fat (fat-poor) are often misdiagnosed as RCC. A Cleveland Clinic report revealed that imaging errors led to the preoperative misdiagnosis of 55% of AML patients as having RCC. Unnecessary procedures, including radical nephrectomies, can be prevented through a more targeted preoperative diagnosis specifically aimed at benign nonlethal tumors [26]. During the development phase, models trained on unenhanced CT images outperformed those using enhanced CT images in fivefold cross-validation, achieving the best patient-level performance, with an average AUC of 0.951. This model demonstrated strong validation results, with AUCs of 0.966 and 0.898 in both internal and external tests, particularly for large tumors (≥40 mm). These findings illustrate the potential of the multichannel deep learning classifier, based on unenhanced whole-tumor CT images, as an effective tool for differentiating RCC from fat-lacking AML [27].

Contrast-enhanced ultrasonography (CEUS) offers real-time visualization of tissue perfusion with outstanding temporal and spatial resolution. Its unique benefits, such as being radiation-free, highly repeatable, and remarkably convenient, have made it a widely preferred tool for kidney examinations. Despite this, texture information is present in the images that the naked eye cannot observe, therefore limiting its diagnostic efficacy. With the intent to successfully predict preoperative degrees of differentiation, researchers developed a radiomic ML model based on renal cancer CEUS images. The results showed an AUC of 0.811, a specificity of 0.786, and an accuracy of 0.784, reinforcing the utility for WHO/ISUP nuclear grading and the non-invasive diagnosis of ccRCC in clinical settings [28].

Yang et al. employed a similar approach to assess the feasibility and efficiency of automatic segmentation of CEUS images in renal tumors by CNN. All seven CNN-based models achieved good performance. The UNet++ model showed the best results, with a mean Intersection over Union (mIOU) of 93.04%, Dice Similarity Coefficient (DSC) of 92.70%, precision of 97.43%, and recall of 95.17% [29].

Ultrasound-based point shear wave elastography remains a topic of debate due to inconsistent findings and limited experience with this imaging diagnostic technique. This controversy can be attributed to various factors, such as the limited depth detection capabilities for deep-seated tumors and vascular disturbances that disrupt shear wave propagation, impacting the accuracy of rigidity measurements [30].

Sagreiya et al. integrated four machine learning algorithms—logistic regression, naïve Bayes, quadratic discriminant analysis, and support vector machines (SVMs)—and evaluated statistical data from the tumor, cortex, and a combination of tumor–cortex–medulla inputs. SVMs showed the highest performance, achieving 94% accuracy and an AUC of 0.98 and significantly outperforming the median SWV. Most models using combined inputs demonstrated good performance, highlighting the algorithms’ ability to differentiate between RCC and AML with high classification accuracy [31].

Magnetic resonance imaging (MRI) provides an alternative diagnostic tool for pregnant women and those with allergies to intravenous contrast agents. It also provides a superior functional outlook when assessing the inferior vena cava compared to contrast CT. Xi et al. implemented a deep learning network using ResNet to analyze MRI data (T1C and T2WI) in order to differentiate between RCC and benign renal masses. The model outperformed both radiomics and expert models in distinguishing RCC [32].

In a related study, Zheng et al. evaluated the efficacy of ResNet using MRI to discriminate between high- and low-grade RCCs in a sample of patients with AJCC grade I and II. The deep CNN model, utilizing T2-weighted fat saturation sequence MR images, showed strong potential for classifying renal parenchymal tumor subtypes. It achieved an overall accuracy of 60.4% and an average accuracy of 61.7%. The macro-average AUC was 0.82, with specific AUCs of 0.94 for ccRCC, 0.78 for chRCC, 0.80 for AML, and 0.76 for pRCC [33].

A summary of the reviewed literature on AI applications in imaging technology for RCC diagnostics is provided in Table 3.

### 3.4. Perioperative Diagnostics

The rapid developments in artificial intelligence technology have significantly facilitated its application in both preoperative and operative procedures. Focusing on advancements in surgical practices, Nakawala et al. sought to optimize the surgical workflow through the Deep-Onto network, which offers essential support in all surgical aspects, including instruments, anatomical considerations, and procedural necessities. Utilizing more than 700,000 frames extracted from nine comprehensive robotic-assisted partial nephrectomy (RAPN) videos, a total of ten surgical phases were delineated, with the data subsequently categorized into test, training, and validation sets. Due to its early implementation, the training models achieved favorable success, demonstrating a precision of 74.0% and an accuracy of 74.3% in predicting the steps of RAPN. According to the authors, AI-driven analysis or intraoperative imaging can provide intraoperative feedback, refine the workflow, and even adapt to unanticipated scenarios [34].

Amir-Khalili et al. sought to reduce the incidence of intraoperative complications by employing an automated guidance system that utilizes advanced diagnostic support to facilitate the identification of hidden accessory vessels, particularly those hidden within fat. The system analyzed temporal motion characteristics in the endoscopic video, where pulsatile motion, along with static visual attributes like texture, intensity, and color, served as new features for vessel segmentation. The AUC for this technique was 0.72, indicating promising initial results [35].

In another study, with a dataset derived from 15 RAPN videos, random decision forest ML programs were enhanced and trained to incorporate data from preoperative imaging studies to estimate and diagnose any three-dimensional anatomical abnormalities. By utilizing preoperative data as patient-specific prior knowledge alongside vasculature pulsation and endoscopic visual cues, the technique achieved a 45% improvement in structure identification. These enhancements enabled precise segmentation within the highly noisy and cluttered environment of endoscopic videos, demonstrating its effectiveness in challenging surgical settings [36].

In rare cases of RCC with a concurrent duplex kidney, Nguyen et al. used Fujifilm’s Synapse AI Platform to visualize the collecting system, kidney, and renal artery system for diagnostic and surgical planning. The Synapse 3D platform generated precise 3D simulations to refine tumor identification and enhance the visualization of the feeding vessels’ spatial relationship. It was noted that AI could facilitate planning for minimally invasive procedures, reduce blood loss, and ensure shorter recovery times. AI could also mitigate inconsistencies associated with traditional manual tracing of CT scans, such as operator-dependent errors and variations in scan timing and contrast conditions. By formatting detailed and objective images through the application of the shape and density model, it helped navigate the intricate vascular and parenchymal architecture within the kidney. The automative technology of Synapse streamlined the creation of vascular maps, diminishing preparation time whilst simultaneously aiding surgical manipulation [37].

Table 4 presents a summary of the reviewed literature on AI applications in perioperative RCC diagnostics.

## 4. Discussion

The integration of AI into contemporary medical practices is still in its nascent stage. Despite the promising outcomes of its current application, patient care and safety remain of utmost importance, allowing no margin for error. AI’s effective and reliable implementation in clinical settings requires further enhancements and an extensive repository of training data.

Within histopathology, numerous challenges and limitations are presented. Nyman et al. noted that the process of tumor evolution was ongoing, whilst the tissue samples were limited to pre-treatment tumor states. The analyzed cells did not align with the ongoing tumor evolution, resulting in a discrepancy between the two samples [8]. When untreated patients are concerned, it is essential to involve larger clinical cohorts using a more diverse and generalized grading approach. To optimize the learning program and enhance the diagnostic workflows, additional histologic features and advanced microheterogeneity observations are necessary. Another limitation of Nyman et al.’s study was the scarcity of pixel-level annotation of all images. Nuclear grading, regarded as essential for histological grading, lacks a definitive and practical framework for the production of consistent annotations, primarily due to the imprecision associated with diverse tumor microenvironment phenotypes [8].

Due to the retrospective nature of the study conducted by Zheng et al., the stratification threshold for the optimal tumor mutation burden should be adjusted if applied to different cohorts, such as the Clinical Proteomic Tumor Analysis Consortium Cohort. The imbalance in sample categories for model training constituted another limitation of the study. The influence of epigenetic modifications, such as methylation, on VHL loss warrants further investigation to better understand their role. By integrating clinical data, multi-omics, and imaging modalities, the overall performance of these frameworks can be upgraded to maximize it [12].

Several challenges associated with inadequately supervised models emerge within the domain of computational pathology. One problem encountered by researchers was the separation of different locations within the slide, causing independent categorizations of regions despite originating from one specimen. In addition, the model may exhibit a deficiency in contextual awareness owing to its inability to learn potential nonlinear interactions among instances. Furthermore, while refining the feature encoder via end-to-end training and utilizing comprehensive data augmentation is expected to improve performance, conducting training directly within the original image pixel space is projected to considerably extend the overall training duration and necessitate increased computational resources [38].

Selection bias presents a significant obstacle to the reliability of outcomes in biomarker discovery. Nevertheless, the implementation of randomized controlled trials in the context of renal cancer diagnostics is unfeasible due to the inherent challenges associated with randomizing interventions for renal cell carcinoma. To address selection bias, Bifarin et al. made adjustments for five confounding variables, including gender, BMI, age, race, and smoking history. Even though the adjustments were effective for four of the factors, continuous research with geographically and racially diverse cohorts of larger sizes is needed for further validation [18].

Radiomic variability associated with the use of multiple CT scanners is discussed in Jaggi et al.’s study. Restricting the analysis to only single-phase CT images significantly hindered the nephrographic phase. With the slice thickness limited to 5 mm, there could be a subsequent reduction in radiomic performance. The use of a single reader for annotation might negatively affect generalizability. Model inflation may arise due to filtering features based on repeated radiomic analyses, emphasizing the need for additional validation to ensure repeatability. The model’s training on a binary classification task limits its application by excluding other renal carcinomas [22].

Similar limitations were reported by Yao et al., where CT images from the nephrographic and excretory phases were excluded from model training, primarily due to the extended period of the cases reviewed. In many of the earlier cases, the image quality of the nephrographic and excretory phases was poorer than that of the corticomedullary phase. This was particularly evident in reconstruction thickness, the clarity of tumor scans, and the scheduling of the scans [27]. In instances where image analysis is the primary objective of artificial intelligence integration, label noise remains a prevalent issue. This phenomenon subsequently undermines the quality of labels associated with medical images, leading to a mismatch with their corresponding actual images. Such discrepancies adversely affect the advancement of deep learning (DL) methodologies.

Another issue that necessitates consideration is the manual filtration of samples, which leads to increased labor expenses and reduced efficiency when handling extensive datasets. In instances of substantial class imbalance, the efficacy of ML and DL models may be compromised, as most classification methods assume an equal likelihood of occurrence for all classes [24]. Mahmud et al. have also raised similar concerns regarding dataset generalizability with the KiTS dataset, thereby underscoring the need for more extensive datasets specifically curated for AI. In order to achieve a balanced representation of tumor stages and subtypes, it is necessary to include individual data from large populations. The performance and reliability of the AI system would be significantly improved through the incorporation of a broader range of clinical biomarkers, comprehensive patient histories, data related to surgical decision-making, and insights into patient behaviors [25].

The automated segmentation of CEUS faces challenges mainly associated with the poor performance of the automatic segmentation algorithm and inconsistencies in the relationship of shape textures. Furthermore, without incorporating CEUS video formatting for automated segmentation, there would be an inadvertent loss of temporal information [29]. In some studies, a single operator performed the majority of the examinations, which poses a problem for the reliability of the results. Nonetheless, previous studies indicate that shear wave elastography measurements show a high degree of reproducibility within the kidney [31].

MR imaging presents a unique set of challenges for segmentation algorithms, particularly in differentiating renal lesions from other parts of the kidney, such as cysts, as well as from other organs with similar intensity. Moreover, differentiating small renal lesions from the surrounding background proves to be difficult. This issue stems from the scarcity of published literature regarding renal lesion identification through advanced neural networks for MR imaging. Furthermore, analyses have been exclusively concentrated on the initial post-contrast phase [32].

## 5. Conclusions

Recently, the diagnostics infrastructure for RCC has undergone major improvements that have made early detection, tumor classification, and treatment planning more accurate and effective. According to the literature review, AI can provide a unique and deep understanding of RCC biology in clinical practice. It can also enhance data-driven diagnostics by integrating with advancements in histology, multi-omics, imaging, and perioperative diagnostics.

In histopathological diagnostics, AI-assisted imaging analysis can help pathologists discover microscopic patterns that are not visible to the human eye, thus enhancing tumor classification and subtyping.

Multi-omics expansion has provided a platform for AI to optimize sequencing analysis. By providing comprehensive molecular profiling, AI paves the way to earlier RCC detection and facilitates a more personalized approach to therapeutics.

AI-assisted differentiation of renal cell carcinoma (RCC) from other renal lesions, as well as in tumor staging, has provided clinicians with useful information. The integration of radiomic features with clinical metadata further enhances the results, enabling clinicians to implement personalized treatment plans.

In perioperative diagnostics, the application of AI can enhance decision-making, improve patient safety, reduce intraoperative complications, and expedite recovery.

Alongside the major advancements in AI-assisted diagnostics, future applications of AI in RCC diagnostics should address the following issues: controlling selection bias, including larger and more diverse datasets, ensuring reliable validation, and advancing AI capabilities for clinical settings. Despite the challenges, using AI to assist with RCC diagnosis could lead to better patient outcomes, a new standard of care for RCC patients, and more personalized cancer management for each patient.

## Figures and Tables

**Table 1:** Summary of the reviewed literature on AI applications in histology for RCC diagnostics.

Study	AI Application	Main Results/Conclusions
Nyman et al. *Cell Rep Med.* 2023 [8]	Deep learning framework using ResNet50 CNN to analyze WSIs of ccRCC.	Identified tumor tissue and nuclear grade, enhancing intratumor heterogeneity analysis beyond manual review. Improved prognosis for immune checkpoint inhibitors in clinical settings.
Holdbrook et al. *JCO Clin Cancer Inform.* 2018 [9]	Automated pipeline for measuring nuclear pleomorphic patterns in ccRCC.	Classified histopathologic images into low or high grade. Strong correlation (r = 0.59) between predicted feature values and regional multigene scores.
Abu Haeyeh et al. *Bioengineering* *(Basel).* 2022 [10]	Deep learning system involving decision-fusion methodologies for renal carcinoma classification.	Achieved 93.0% accuracy, 91.3% sensitivity, and 95.6% specificity in distinguishing ccRCC from other renal cell carcinoma subtypes. Outperformed ResNet-50 model.
Abdeltawab et al. *Sci Rep.* 2021 [11]	Pyramidal deep learning pipeline with three CNNs for kidney WSIs.	Enhanced pathologists’ accuracy in automated histopathological diagnosis. Outperformed other neural networks, including ResNet.
Zheng et al. *Cancer Med.* 2024 [12]	Self-supervised attention-based multiple instance learning (SSL-ABMIL) for tumor mutation burden and VHL mutation status.	The SSL-ABMIL model was found effective in forecasting VHL and TMB mutations based on histological characteristics and found to be capable of integrating molecular biology with tumor morphology.
Cai et al. *Biomed Res Int.* 2022 [13]	Integration of texture feature descriptors with deep learning models.	The highest accuracy (98.54%) was achieved using an SVM classifier with an AlexNet + Gabor filter, followed by AlexNet + MRF (97.39%). Other texture combinations also demonstrated high accuracy, exceeding 90.0%.
He et al. *Sci Rep.* 2020 [14]	KNN machine learning applied to immunohistochemical (IHC) images of RCC tissue microarrays.	Identified LC3B as a strong ccRCC marker. Demonstrated p62 as a key marker for differentiating chromophobe RCC. Enhanced precision oncology applications.

**Table 2:** Summary of the reviewed literature on AI applications in multi-omics technology for RCC diagnostics.

Study	AI Application	Main Results/Conclusions
Jagga et al. *BMC Proc.* (2014) [17]	Supervised learning algorithms (J48, RF, SMO, Naive Bayes)	Differentiated between early- and late-stage ccRCC using transcriptomic signatures. The random forest (RF) model performed best, achieving an AUC of 0.80, 89% sensitivity, and 77% accuracy. Identified 62 genes that differentiated between the early- and late-stage groups.
Liu et al. *Biomed Res Int.* 2020 [17]	Bioinformatics in neural networks	Identified hub genes (TPX2, AURKB, NCAPG, CCNA2) as significant biomarkers for ccRCC.
Bifarin et al. *J Proteome Res.* 2021 [18]	Machine learning system using multiplatform urine-based metabolomics	Demonstrated the potential of machine learning in non-invasive RCC detection, improving diagnostic accuracy: AUC = 0.98, accuracy = 88%, sensitivity = 94%, specificity = 85%.
Manzi et al. *J Proteome Res.* 2021 [20]	Machine learning approach combined with mass spectrometry-based lipidomics	Identified lipid panels to differentiate ccRCC from controls and early/late-stage ccRCC. The 16-lipid panel: 95.7% accuracy (training), 77.1% accuracy (testing); early/late-stage differentiation: 82.1% accuracy.
Iwamura et al. *Cancer Sci.* 2022 [21]	AI-optimized liquid biopsy (N-glycan signatures)	Demonstrated the potential of glycan-based biomarkers for precision diagnosis. Scoring system for RCC probability using serum data: AUC = 0.99, sensitivity = 90%, specificity = 99%.
Jaggi et al. *J Med Imaging (Bellingham)* (2021) [22]	Random Forest, AdaBoost Classifiers on radiomic features	Demonstrated the potential of radiomics in improving non-invasive tumor classification. Feature clustering revealed seven distinct clusters in each radiomic biopsy (RB) round with Rand index = 0.981.
Brennan et al. *JCO Precis Oncol* (2020) [23]	Machine learning to identify signatures of differentially methylated cytosine–phosphate–guanine sites (CpGs)	Differentiated oncocytoma from chromophobe RCC using DNA methylation signatures: AUC = 0.96 (oncocytoma vs. chRCC); AUC = 0.87 (chRCC vs. other RCC subtypes).

**Table 3:** Summary of the reviewed literature on AI applications in imaging technology for RCC diagnostics.

Study	AI Application	Main Results/Conclusions
Xu et al. *Cancers (Basel)* 2022 [24]	Stochastic gradient descent (SGD) algorithm with cross-entropy loss was used to pre-train deep learning models. Ensemble model was applied to 9978 images.	The ensemble model achieved superior performance (AUC = 0.882) compared to the single model (AUC = 0.864). AI demonstrated potential for non-invasive RCC diagnosis.
Mahmud et al. *Cancers (Basel)* 2023 [25]	A machine learning approach was used to analyze CT scans and clinical data in order to grade tumors and determine the most suitable surgical procedure.	Cancer grading showed 85.66% accuracy, 84.18% precision, and 84.92% F1-score. Surgical selection demonstrated 90.63% accuracy, 90.83% precision, and 90.50% F1-score. Tumor volume and stage were identified as key factors.
Yao et al. *J Cancer Res Clin Oncol.* 2023 [27]	A multichannel deep learning model was applied to unenhanced CT images for differentiating RCC from fat-lacking angiomyolipoma.	Best performance with unenhanced CT (AUC = 0.951); validation results (AUC = 0.966 internal, 0.898 external) were particularly effective for large tumors (≥40 mm).
Luo et al. *BMC Med Imaging* 2024 [28]	Machine learning was applied to contrast-enhanced ultrasound (CEUS) images to develop a radiomic model for RCC diagnosis.	Achieved AUC of 0.811, specificity of 0.786, and accuracy of 0.784. Reinforced the utility of WHO/ISUP nuclear grading for non-invasive CCRCC diagnosis.
Yang et al. *Front Oncol.* 2023 [29]	Deep learning CNN models for automatic segmentation of CEUS images in renal tumors.	The UNet++ model performed the best, yielding a mean Intersection over Union (mIOU) of 93.04%, Dice Similarity Coefficient (DSC) of 92.70%, precision of 97.43%, and recall of 95.17%.
Sagreiya et al.* Ultrasound Med Biol.* 2019 [31]	Integrated four machine learning algorithms, including logistic regression, naïve Bayes, quadratic discriminant analysis, and support vector machine (SVM), to evaluate tumor–cortex–medulla input data.	SVM achieved the highest performance, with an accuracy of 94% and an AUC = 0.98. Demonstrated strong differentiation between RCC and AML.
Xi et al. *Clin Cancer Res.* 2020 [32]	ResNet deep learning network applied to MRI (T1C and T2WI) for differentiating RCC from benign renal mass.	Outperformed radiomics and expert models in distinguishing RCC. Demonstrated AI-enhanced MRI diagnosis.
Zheng et al. *Abdom Radiol (NY).* 2021 [33]	ResNet was applied to MRI (T2-weighted fat saturation sequence) to discriminate high- from low-grade RCCs.	The model achieved overall accuracy of 60.4% and macro-average AUC of 0.82 with specific AUCs of 0.94 for ccRCC, 0.78 for chRCC, 0.80 for AML, and 0.76 for pRCC.

**Table 4:** Summary of the reviewed literature on AI applications in perioperative RCC diagnostics.

Study	AI Application	Main Results/Conclusions
Nakawala et al. *Int J Comput Assist Radiol Surg.* 2019 [34]	Deep-Onto network applied to robotic-assisted partial nephrectomy (RAPN) workflow using 700,000+ frames from nine RAPN videos.	Identified ten surgical phases, with the data categorized into test, training, and validation sets. This early model achieved 74.0% precision and 74.3% accuracy in predicting RAPN steps.
Amir-Khalili et al. *Med Image Anal.* 2015 [35]	AI-guided intraoperative imaging system using motion characteristics for hidden vessel identification.	Identified hidden accessory vessels with an AUC of 0.72, demonstrating potential for reducing intraoperative complications.
Nosrati et al. *Int J Comput Assist Radiol Surg.* 2016 [36]	Machine learning models were trained on 15 RAPN videos to integrate preoperative imaging for anatomical abnormality detection.	Improved structure identification by 45%, enabling precise segmentation in noisy endoscopic environments.
Nguyen et al. *J Med Case Rep.* 2024 [37]	Fujifilm Synapse AI Platform was used to generate 3D simulations for RCC cases with duplex kidneys.	Enhanced tumor visualization and vascular mapping, reducing preparation time and blood loss and aiding minimally invasive surgical planning.

## Data Availability

Not applicable.

## Abbreviations

The following abbreviations are used in this manuscript:

AI	Artificial intelligence
RCC	Renal cell carcinoma
ccRCC	Clear cell renal cell carcinoma
chRCC	Chromophobe renal cell carcinoma
ccpRCC	Clear cell papillary renal cell carcinoma
non-RCC	Non-renal cell carcinoma
RMB	Renal mass biopsy
RB	Radiomic biopsies
ML	Machine learning
CNN	Convolutional neural network
TMB	Tumor mutation burden
